# Bioinformatic analyses and experimental validation of the role of m6A RNA methylation regulators in progression and prognosis of adrenocortical carcinoma

**DOI:** 10.18632/aging.202896

**Published:** 2021-04-21

**Authors:** Fangshi Xu, Yibing Guan, Yubo Ma, Li Xue, Peng Zhang, Xiaojie Yang, Tie Chong

**Affiliations:** 1 Department of Medicine, Xi’an Jiaotong University, Xi’an, Shaanxi 710061, China; 2 Department of Urology, The Second Affiliated Hospital of Xi’an Jiaotong University, Xi’an, Shaanxi 710000, China

**Keywords:** adrenocortical carcinoma, N6-methyladenosine, methylation, prognosis, risk signature

## Abstract

M6A-related genes have been proven to play an important role in many cancers. However, the role of that in adrenocortical carcinoma (ACC) has not been fully elucidated. In the present study, 77 ACC samples from TCGA database were divided into localized (*n* = 46) and metastatic (*n* = 31) groups. Three differential expression genes (DEGs) and five prognostic m6A genes were screened out. M6A-related risk signature (RBM15 and HNRNPC) was constructed by the Lasso regression analysis. In TCGA cohort (training cohort), the risk signature was identified as an ACC-independent prognostic factor and can distinguish the prognostic difference of ACC patients with clinical stage I-II, T3-4 and N0 stages. A nomogram combining T stage and m6A risk score was constructed to predict the overall survival rate (OSR) of individual at 1,2,3 year. Meanwhile, its prognostic value was also confirmed in the validation cohort (GSE33371 dataset). The potential associations between m6A risk level and immune checkpoint inhibitors (ICIs) therapy were also investigated via the TISIDB online tool. High m6A risk not only can suppress immunotherapy-related biological processes, but also repress the expressions of immune-checkpoint markers. Moreover, five pairs of clinical specimens were collected to confirm the overexpression of HNRNPC and non-ectopic expression of RBM15 in tumor tissues. HNRNPC was proven to promote the proliferation, migration and invasion of H295R and SW13 cells through MTT and Transwell assays. In conclusion, the m6A-related risk signature was beneficial for prognostic analysis and can affect immune microenvironment in ACC. HNRNPC played a pro-cancer role in ACC progression.

## INTRODUCTION

Adrenocortical carcinoma (ACC) is a rare malignant tumor with an incidence rate of approximately 0.7/million to 2.0/million in the population [1]. ACC is characterized by its high malignancy, fierce invasiveness and early metastasis, which leads to a poor prognosis. The overall survival (OS) time of ACC patients is only 4 to 30 months, and the 5-year overall survival rate fluctuates from 16% to 47%. In addition, approximately 60% of ACC patients reach the advanced stage at the time of diagnosis, and the 5-year OS rate of these patients plummets to 5%-10% [2]. Despite the persistent improvement of the therapeutic methods and concepts of ACC, the amelioration of the OS rate is still limited [3]. Even some classic molecular targeted drugs, such as IGF1R (insulin-like growth factor 1 receptor) inhibitors [4], VEGF (vascular endothelial growth factor) inhibitors [5] and EGFR (epidermal growth factor receptor) inhibitors [6], have been proven to fail to effectively prolong the OS time of ACC patients. Based on the severe therapeutic dilemma of ACC, some researchers even pessimistically asserted that the curative effect of ACC may not make a breakthrough in 10 to 15 years [7]. Therefore, it is crucial, urgent and challenging to elaborate the molecular mechanisms of ACC carcinogenesis and progression.

Among kinds of epigenetic modifications, N6-methyladenosine (M6A) is the most common form of RNA modification, accounting for approximately 60% of all RNA modifications, and it can edit all types of RNA [8]. M6A RNA methylation can appear in 0.1% to 0.4% of total adenosine residues [9], and involves the mRNA regulation of more than 7600 genes [10]. Therefore, m6A RNA methylation is the major method of posttranscriptional regulation and contributes to the regulation of mRNA stability, alternative splicing, intracellular distribution and transcription [11]. The process of m6A is mediated by three groups of genes, namely, ‘Writer’, ‘Eraser’ and ‘Reader’. The ‘Writer’ genes (including KIAA1429, METTL3, METTL14, RBM15, WTAP and ZC3H13) can transfer the methyl group to the nitrogen on the sixth carbon of the aromatic ring of an adenosine residue [12]. Through methylation modification, the protein expression of the target mRNA is decreased. ‘Eraser’ genes (including ALKBH5 and FTO) are responsible for the reversion of methylation [13]. The ‘reader’ genes (including HNRNPC, YTHDC1, YTHDC2, YTHDF1 and YTHDF2) are effectors that decode the m6A methylation information and transform it into a functional signal [14].

M6A regulatory genes have been proven to be involved in the carcinogenesis and progression of various tumors [12]. The downregulation of METTL3 and METTL14 can induce the apoptosis and differentiation of acute myeloid leukemia (AML) cells [15, 16]. The aberrant expression of FTO and ALKBH5 promotes the proliferation of glioblastoma by increasing the expression level of FOXM1 [17]. In breast cancer (BRC), elevated METTL3 can promote tumor progression by inhibiting the tumor suppressor let-7g [18]. Although some research has explored the function of m6A regulatory genes in some cancers, such as renal cancer [19], pancreatic adenocarcinoma [20] and hepatocellular carcinoma [21], the role of that in ACC has not been fully elucidated. Therefore, we performed bioinformatic analyses for expression, prognostic value and immunological effect of m6A-related genes in ACC by using TCGA, GEO and GEPIA databases. Further, we validated that HNRNPC could promote the proliferation and invasion of adrenal cancer cells through *in vitro* experiment. In a word, the present study can provide new insights into the pathogenesis and prognosis of ACC.

## MATERIALS AND METHODS

### Data source

The training cohort was obtained from The Cancer Genome Atlas (TCGA) database (https://www.cancer.gov/tcga), including the gene expression data from 79 samples and the clinical data from 90 samples. The type of gene expression data was set as ‘transcriptome profiling’ and ‘gene expression quantification’. The type of clinical data was ‘BCR-XML’. To avoid the interference caused by other special tumor subtypes, the histologic types of ACC were selected as adenomas and adenocarcinomas. The specific database filtering settings were shown in Supplementary Table 1.

To find the appropriate validation cohort, we used ‘adrenal cancer’ as the searching term of GEO database and set ‘Expression profiling by array’ and ‘Homo sapiens’ as the retrieval conditions. Then, a total of 43 datasets were obtained. Among that, 12 datasets relate to ACC cell lines, 3 datasets relate to children ACC, 2 datasets relate to preclinical models, 2 datasets relate to lung metastatic tumors and 4 datasets relate to adrenal hyperplasia or Cushing's syndrome, which were both excluded. Among remaining 20 ACC datasets, only four datasets (GSE19775, GSE19750, GSE19776 and GSE33371) contain clinical information. However, GSE19775 and GSE19776 do not record the survival status, and the survival status of all samples in GES19750 is dead. Therefore, due to absent clinical information or unknown survival outcomes in other GEO datasets, we finally selected GSE33371 as the validation cohort, which contained 33 ACC samples [22]. Among that, 10 samples were excluded for their survival information was unknown and remaining 23 ACC samples were used for the validation of m6A prognostic model.

### Clinical samples

5 pairs of ACC specimens and normal adjacent tissues were collected at department of urology, the second affiliated hospital of Xi'an Jiaotong University (Xi’an, China). All patients submitted informed consent for tissue use. The study protocol was approved by the Ethics Committees of the second affiliated hospital of Xi'an Jiaotong University.

### Selection of m6A-related genes

There are three categories of m6A-related genes: ‘Writer’ genes including KIAA1429, METTL3, METTL14, RBM15, WTAP and ZC3H13; ‘Eraser’ genes including ALKBH5 and FTO; and ‘Reader’ genes including HNRNPC, YTHDC1, YTHDC2, YTHDF1 and YTHDF2. These 13 genes were chosen as the research objects in this study, which was consistent with the strategy adopted in previous studies [22, 23]. Besides, the correlation analysis among m6A-related genes was performed by the ‘corrplot’ package of R software, which is based on the calculation of Spearman's correction coefficient.

### Identification of prognostic m6A-related DEGs

We performed the extraction and arrangement of TCGA data via Perl (Practical Extraction and Report Language) version 5.28. Combined with TCGA clinical information, 77 ACC samples were divided into localized group (*n* = 46) and metastatic group (*n* = 31). The other 2 ACC samples were excluded for their corresponding clinical information was unknown. According to 7^th^ AJCC TNM-stage system for ACC, patients with clinical stage I-II were classified as localized tumors (no extra-adrenal invasion), and those with clinical stage III-IV were defined as metastatic tumors (Lymphatic invasion or distant metastasis). The differentially expressed genes (DEGs) between localized and metastatic groups were determined by the ‘Limma’ package of R software.

Cox univariate regression analyses were employed to screen out the m6A-related genes with the ability to affect ACC prognosis, namely prognostic genes. Additionally, the GEPIA web server (http://gepia.cancer-pku.cn/) [23] was applied to perform overall survival (OS) analysis based on gene expression data from the TCGA and the GTEx projects, which reconfirmed the m6A prognostic genes. Finally, the intersection genes (the prognostic m6A-related DEGs) between DEGs and prognostic genes were identified via the Venn graph. These crossed genes were attempted to construct a m6A-related risk signature.

### Establishment of m6A-related risk signature

We used ‘glmnet’ package to conduct the lasso regression analysis to establish a m6A-related risk signature. According to the prognostic model, the risk score of each sample was calculated, and all ACC samples were divided into high- and low-risk groups based on the median of risk score.

### Survival analyses in training cohort

To evaluate the effect of m6A-related risk signature on ACC prognosis, we compared the prognostic difference between high- and low-risk groups. Subsequently, ROC curves, PCA (Principal component analysis) and t-SNE (t-distributed stochastic neighbor embedding) were used to assess the prediction accuracy of this prognostic model. Decision curve analysis (DCA) was conducted to evaluate the clinical net benefit of m6A risk signature. The relationships between the risk signature and clinicopathological features were displayed by a heatmap.

Moreover, based on multivariate Logistic regression analysis, we constructed a nomogram combining ACC clinicopathological features and m6A risk signature to predict the overall survival rate (OSR) of individual at 1,2,3 year. And the prediction accuracy of the nomogram was estimated via the calibration curve.

Besides, to confirm whether risk score could be an independent prognostic factor in ACC, we performed cox univariate and multivariate analyses successively. In order to assess the application scope of m6A risk signature in ACC prognosis analysis, we compared the prognostic difference between different risk groups under the same clinical subgroups.

### Survival analyses in validation cohort

The m6A prognostic model was tested in the validation cohort (GSE33371). According to the cutoff value calculated with the same prognostic model, 23 ACC samples in validation cohort were divided into high- and low-risk groups. Likewise, the prognostic difference between two groups was compared and ROC curves, independent prognostic analyses were also performed. The distribution of survival outcomes of each ACC sample was displayed using the risk plots. Besides, the heatmap of clinical features and gene expression were drawn by the ‘pheatmap’ package in R software.

### Immune analyses

The immune abundances of 22 leukocyte subtypes in each ACC sample were calculated by the CIBERSORT algorithm [24]. The active levels of 13 immune-related pathways were assessed based on single-sample gene set enrichment analysis (ssGSEA), which was performed by the ‘gsva’ package of R software [25]. And then, the differences of immune infiltration levels and immune pathway activities between high- and low-risk groups were determined by the ‘Limma’ package, which revealed the effect of m6A risk signature on ACC immune microenvironment.

Further, we investigated the potential links between the m6A risk genes and immune checkpoint inhibitor (ICIs) therapy. The expression correlations between two cell-surface mediators (PD-L1 and CTLA4) and m6A risk genes were assessed using TISIDB online tool (http://cis.hku.hk/TISIDB/) [26]. Meanwhile, the impact of PD-L1 (CD274) and CTLA4 expressions on ACC prognosis, and their expressive difference between high- and low-risk groups were also ascertained. Besides, the distribution of HNRNPC and RBM15 expression across different immune subtypes were presented via TISIDB tool.

### Mutation analyses

The cBioPortal database (http://cbioportal.org) provides multidimensional cancer genomics data [27]. Using ‘OncoPrint tab’ and ‘cancer types summary tab’, we summarized the genetic alteration, mutation type and frequency of m6A risk genes.

### Cell culture

Two adrenocortical cancer cell lines, H295R and SW13, were purchase from Institute of Biochemistry and Cell Biology at the Chinese Academy of Sciences (Shanghai, China). H295R cells were cultured in DMEM/F12 (Dulbecco's Modified Eagle Medium) with multiple ingredients including 6.25ug/ml insulin, 6.25ug/ml transferrin, 6.25 ng/ml Selenium, 1.25mg/ml BSA, 5.35 ug/ml Linoleicacid, 2.5% Nu-Serum I and 1% Penicillin-Streptomycin (P/S) (Yipu Biotechnology co, Wuhan, China). SW13 cells were cultured in DMEM added 10% FBS (Fetal bovine serum) and 1% P/S (Yipu Biotechnology co, Wuhan, China). All cells were incubated at 37°C in a humidified atmosphere with 5% CO_2_ and 95% humidity.

### RNA extraction and RT-qPCR

The expressions in mRNA level of HNRNPC and RBM15 in tissues and cells were assessed by RT-qPCR. Clinical tissues were immersed in RNA wait solution (Hengya Biotechnology co, Shanghai, China) at 4°C for 24 h then saved at -80°C. Total RNA were extracted from tissues and cells through TRIzol reagent (G-Clone, Beijing, China). cDNA was synthesized by using RT (Reverse Transcription) reagent Kit (Takara, Japan). qPCR was amplificated via SYBR-Green reagent (Takara, Japan). Expression levels were normalized to GAPDH and the relative mRNA levels were calculated based on 2^−ΔΔCt^ method. Primer list was presented in Supplementary Table 2.

### Cell transfection

Specific small interfering RNAs (siRNAs) were synthesized to inhibit HNRNPC expression and pcDNA 3.1 was employed to carry HNRNPC overexpression sequence. si-HNRNPC and pc-HNRNPC were designed by GenePharma Biotechnology (Shanghai, China). The siRNA sequences were listed in Supplementary Table 2. Lipofectamine 2000 (Invitrogen, Thermo Fisher, Waltham, MA, USA) was used to transfect H295R and SW13 cells. The transfection efficiency was determined by Western blot after cultivation for 48 h.

### MTT assay

MTT assay was performed to evaluate the proliferative ability of adrenal cancer cells. Six groups ACC cells were seeded in 96-well culture plates with the concentration of 5 × 103/well. After incubating for 24 h, 48 h and 72 h, cells were added with MTT Reagent (Solarbio Life Science co, Beijing, China) and cultured for 4 h, at 37°C. Then, DMSO was added in each well after discarding the medium. The absorbance was measured by a microplate reader (ThermoFisher, Waltham, MA, USA) at 490 nm.

### Transwell migration and invasion assay

The migrative and invasive abilities of ACC cells was measured via Transwell chamber (Corning, NY, USA). When conducting invasion assay, 100μL DMEM-diluted Matrigel (Corning, NY, USA) was added to each well before seeding cells. But no Matrigel was added during migration assay. Transfected cells (5 × 10^4^/well) were seeded in upper chamber with serum-free medium and complete DMEM containing 20% FBS was added in the lower chamber. After 48 h incubation and discarding the medium of upper chamber, cells on the upper surface of membrane were removed by twice PBS washing and cotton swab wiping out. The cells adhering to the lower surface of membrane were fixed in paraformaldehyde for 20 minutes and stained by 0.1% crystal violet for 20 minutes. The migrative and invasive cells were observed under a microscope.

### Western blot

Well-proliferative cells were lysed by RIPA (ThermoFisher, Waltham, MA, USA). Protein concentration was detected using BCA Protein Assay Reagent (Pierce, Appleton, Wisconsin, USA). Then, 50 μg protein samples were separated by 10% SDS-PAGE and transferred to nitrocellulose membranes. After blocking the membranes with 10% fat-free milk for 2 h at room temperature, the membranes were incubated with primary antibodies against HNRNPC (HPA051075, Sigma, Louis, MO, USA) at 4°C overnight. After membranes washing, the HRP secondary antibody (goat anti-rabbit IgG, ThermoFisher, Waltham, MA, USA) was added and incubated for 2 h at room temperature.

### Statistical analysis

All statistical analyses were performed using R software (version 3.6.2). The Kolmogorov–Smirnov test was used to analyze the relationships between m6A-related risk signature and the clinicopathological features of ACC. The difference in overall survival rate (OSR) between different groups was compared based on the Kaplan–Meier method. All statistical results with a *p*-value <0.05 were considered as significant.

### Ethics approval and consent to participate

The study protocol was approved by the Ethics Committees of the second affiliated hospital of Xi'an Jiaotong University. All patients submitted informed consent for tissue use.

### Availability of data and materials

The datasets used and/or analyzed in the current study are available from the corresponding author upon reasonable request.

## RESULTS

**Figure 1:**
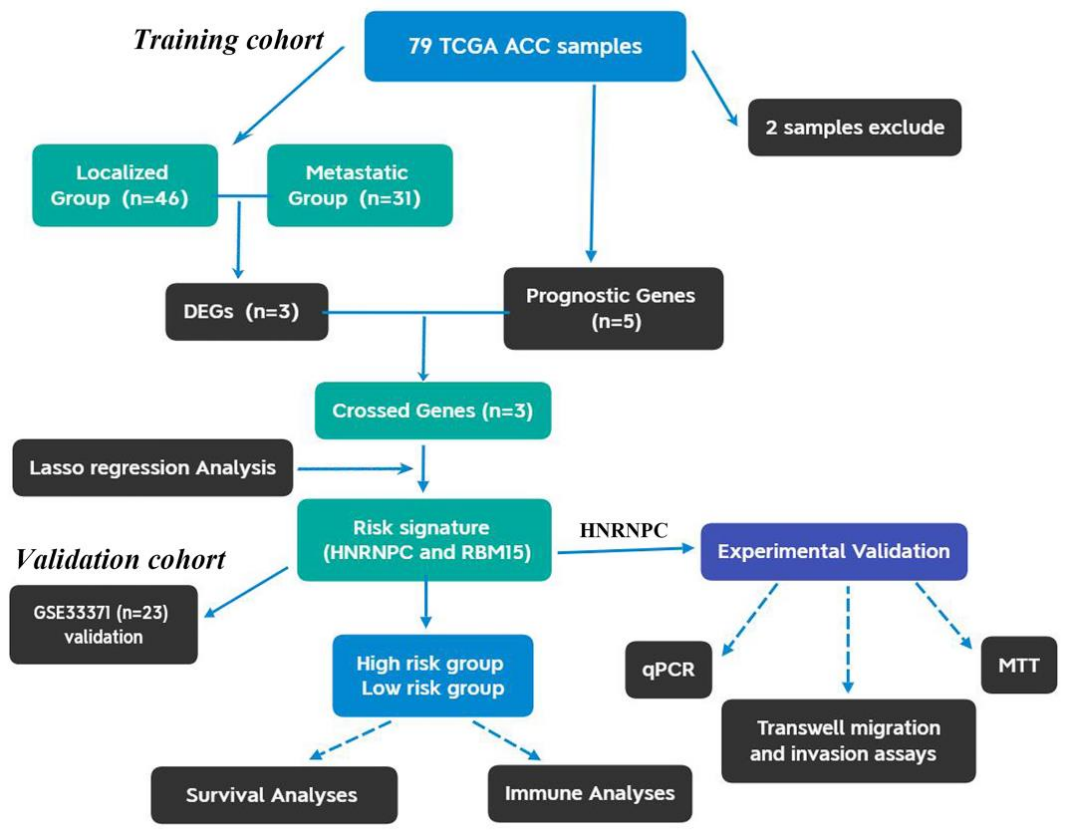
**The flow chart of present study.** ACC, Adrenocortical carcinoma; DEGs, differentially expressed genes.

The flow chart of this study was shown in Figure 1. Using 77 ACC samples from TCGA database, an m6A-related prognostic model was constructed by the Lasso regression analysis. Then, the prognostic model was tested in validation cohort (GSE33371). The clinical characteristics of training and validation cohorts were shown in Tables 1 and 2, respectively. Further, the expression and biofunction of HNRNPC were verified by qPCR, MTT, Transwell migration and invasion assays.

**Table 1:** Clinical characteristics of 77 ACC patients from TCGA database.

**Variables**	**Number (percentage)**
Vital status	
Alive	67 (87.0%)
Dead	10 (13.0%)
Age	NA
Gender	
Male	29 (37.7%)
Female	48 (62.3%)
Tumor Grade	NA
Clinical Stage	
Stage I	9 (11.7%)
Stage II	37 (48.1%)
Stage III	16 (20.7%)
Stage IV	15 (19.5%)
T stage	
T1	9 (11.7%)
T2	42 (54.5%)
T3	8 (10.3%)
T4	18 (23.5%)
M stage	NA
N stage	
N0	68 (88.3%)
N1	9 (11.7%)

Abbreviations: ACC, Adrenocortical carcinoma; NA, not available.

**Table 2:** Clinical characteristics of 23 ACC patients from validation cohort.

**Variables**	**Number (percentage)**
Vital status	
Alive	7 (30.4%)
Dead	16 (69.6%)
Age	
≤65	21(91.3%)
>65	2 (8.7%)
Gender	
Male	7 (30.4%)
Female	16 (69.6%)
Tumor diameter	
>10 cm	12 (52.2%)
≤10 cm	7 (30.4%)
Unknown	4 (17.4%)
Tumor weight	
>500 mg	8 (34.8%)
≤500 mg	8 (34.8%)
Unknown	7 (30.4%)
Clinical Stage	
Stage I	2 (8.7%)
Stage II	10 (43.5%)
Stage III	3 (13.0%)
Stage IV	8 (34.8%)
Weiss score	
High	17 (73.9%)
Low	6 (26.1%)
T, M and N stages	NA

Abbreviations: ACC, Adrenocortical carcinoma; NA, not available.

### Thirteen regulatory genes play a crucial role in m6A methylation

**Figure 2:**
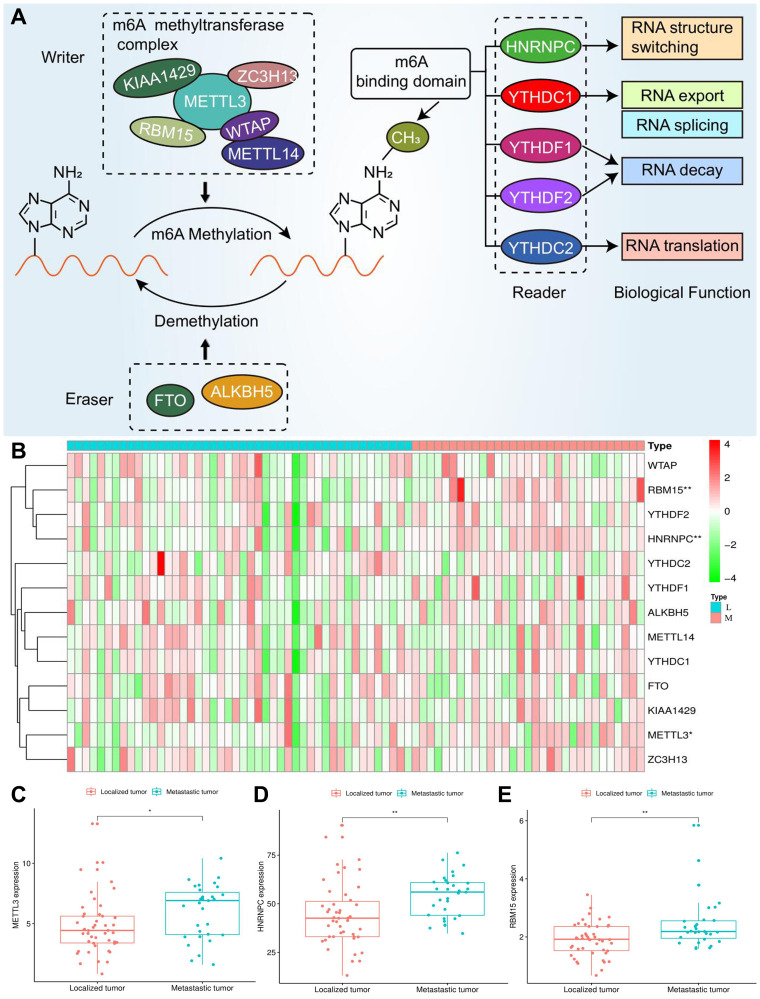
**Expression of m6A regulators in ACC.** (**A**) The process and molecular functions of m6A RNA methylation. (**B**) The expressive heatmap of m6A-related genes. The gene symbols are on the right of the heatmap. L, localized tumor (*n* = 46). M, metastatic tumor (*n* = 31). High expression is shown in red and low expression is green. (**C**–**E**) Differential expression of METTL3, HNRNPC and RBM15 between localized and metastatic tumor samples. Gene expression is measured by FPKM. FPKM, Fragments Per Kilobase per Million. ^*^P < 0.05, ^**^P < 0.01.

Three types of m6A regulatory genes were included in the subsequent bioinformatic analyses. The functions of these regulatory genes and the process of m6A methylation were shown in Figure 2A. A multicomponent methyltransferase complex consisting of ‘Writer’ genes is responsible for m6A methylation. METTL3 and METTL14 constitute the core portion of m6A complex, in which only METTL3 possesses the activity of methyltransferase, while, METTL14 provides a structural support for RNA binding [28]. WTAP acts as an adaptor protein to bridge METTL3 and METTL14 [29]. Besides, other ‘Writer’ genes (KIAA1429, RBM15 and ZC3H13) are proven to serve as auxiliary subunits required for m6A methylation. ‘Eraser’ genes, FTO and ALKBH5 have the ability of demethylase, therefore, which can reverse the m6A methylation [30]. All bioproducts of ‘Reader’ genes can recognize and bind to the m6A-modified sites on RNA. These genes regulate mRNA abundance through affecting RNA splicing, transport, translation and changing structural stability of RNA [28].

### Some m6A-related genes are differential expressive in metastatic ACC samples.

The expression differences of m6A genes between localized and metastatic ACC samples were shown in Figure 2B–2E. A violin plot of differential expression of m6A genes was presented in Supplementary Figure 1. Among 13 m6A regulators, only METTL3, HNRNPC and RBM15 were upregulated in metastatic ACC samples, which suggested that these m6A DEGs may serve as oncogenes in ACC progression. Besides, the correlations among m6A genes were presented in Supplementary Figure 2. All regulatory genes displayed positively co-expression and METTL14-YTHDC1 possessed the largest correlation coefficient (0.67).

### Screening the m6A genes associated with ACC prognosis

**Figure 3:**
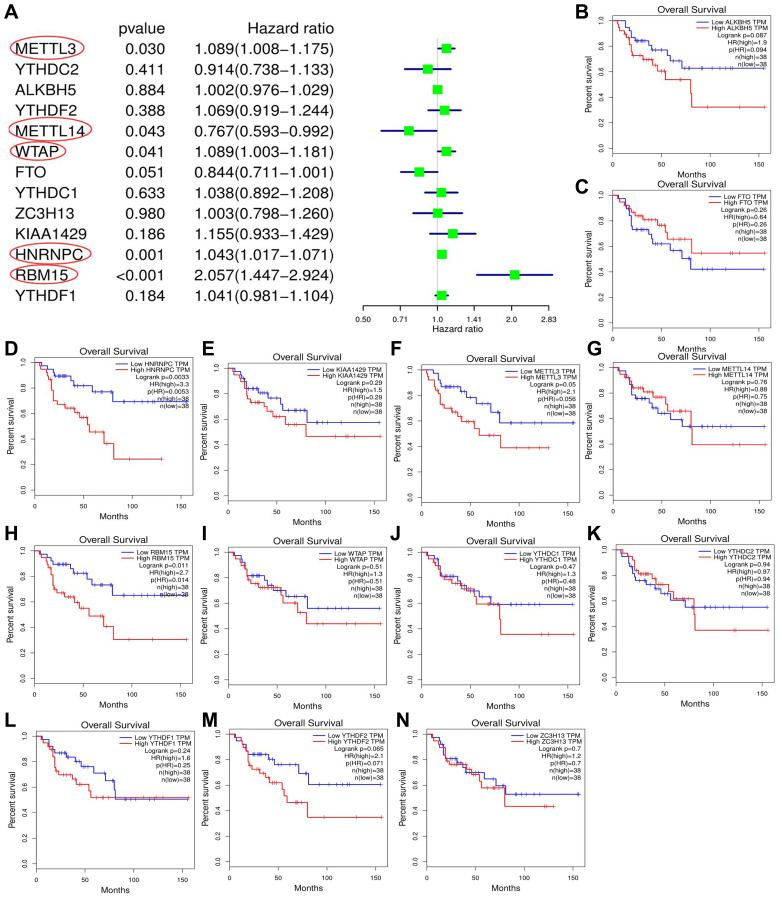
**Screening of m6A prognostic genes.** (**A**) Cox univariate prognostic analyses of m6A-related genes based on TCGA database. (**B**–**N**) Prognostic difference of m6A-related genes between high- and low-expression groups based on GEPIA database. Group cutoff is set as median of gene expression.

Through cox univariate analysis of TCGA data, 5 genes were proven to be related to ACC prognosis, namely METTL3 (*p* = 0.030, HR = 1.089), METTL14 (*p* = 0.043, HR = 0.767), WTAP (*p* = 0.041, HR = 1.089), HNRNPC (*p* = 0.001, HR = 1.043) and RBM15 (*p <* 0.001, HR = 2.057) (Figure 3A). Moreover, we reconfirmed the m6A prognostic genes via GEPIA database (Figure 3B–3N). Similar with TCGA analytical results, METTL3 (*p* = 0.05, HR = 2.1), HNRNPC (*p* = 0.0033, HR = 3.3) and RBM15 (*p <* 0.011, HR = 2.7) can still affect ACC survival outcomes (Figure 3D, 3F, 3H). However, the ectopic expression of METTL14 and WTAP did not bring a prognostic difference (Figure 3G–3I).

### Construction and assessment of m6A-related risk signature.

**Figure 4:**
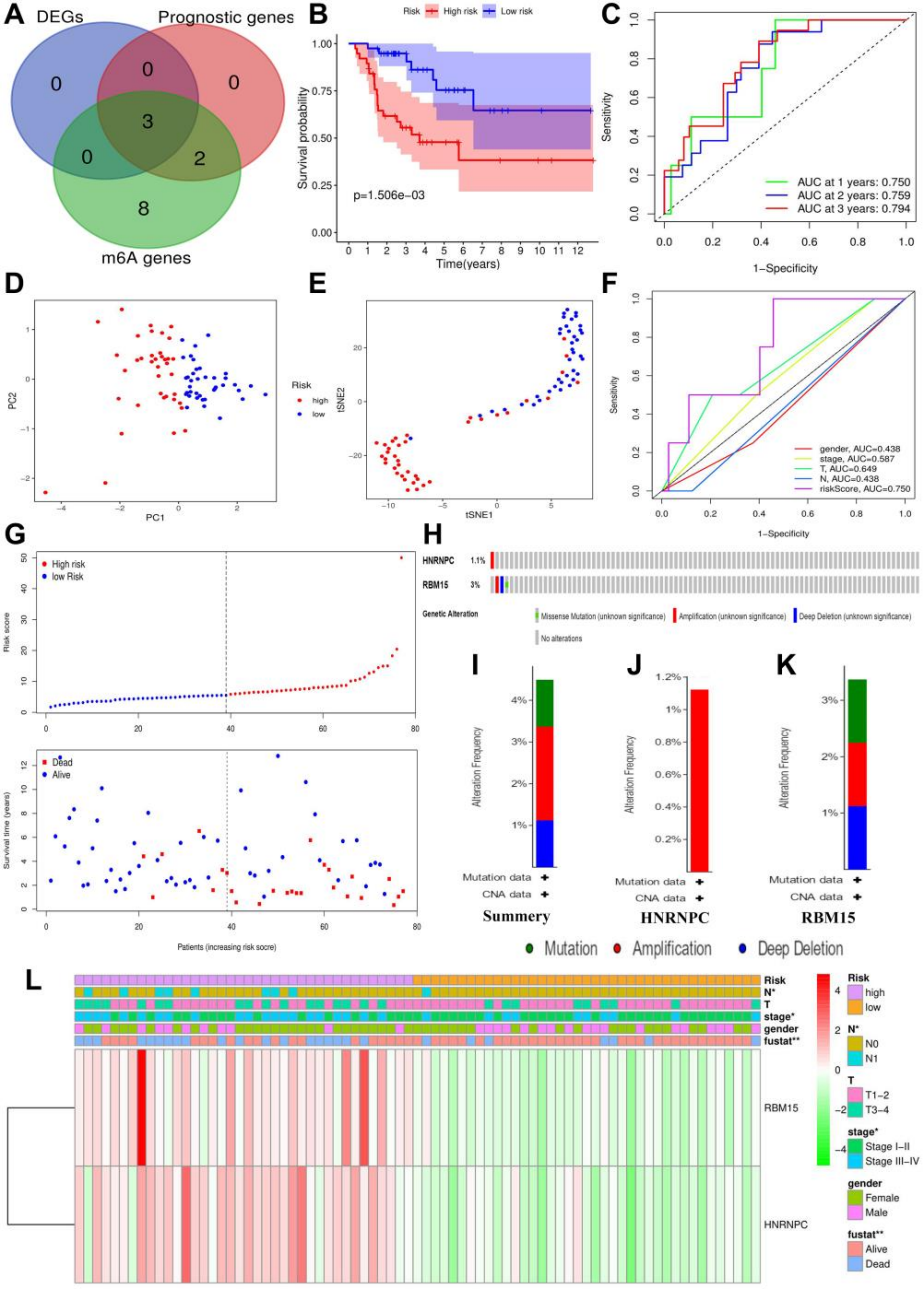
**Construction of m6A-related risk signature.** (**A**) Identification of m6A-related prognostic DEGs. (**B**) The survival difference between the high- (*n* = 39) and low-risk groups (*n* = 38). (**C**) Time-dependent ROC curves of m6A risk signature. (**D**) PCA plot of m6A risk signature. (**E**) t-SNE plot of m6A risk signature. (**F**) ROC curves of m6A risk signature and ACC clinical parameters. (**G**) Risk plots of m6A risk signature. (**H**) The summary of HNRNPC and RBM15 mutations. (**I**) The summary of mutation types. (**J**) The mutation types of HNRNPC. (**K**) The mutation types of RBM15. (**L**) The heatmap of m6A risk signature. The relationships between m6A-related risk levels and the clinicopathological characteristics of ACC were determined by Kolmogorov–Smirnov test. Overexpression is presented in red and low expression is green. DEGs, differentially expressed genes; ROC, receiver operating characteristic curve; PCA, principal component analysis; s-SNE, t-distributed stochastic neighbor embedding; ^*^P < 0.05, ^**^P < 0.01.

According to the above analyses, we finally identified three m6A genes with both discernable expression and affecting prognosis via Venn diagram (Figure 4A). Subsequently, these m6A prognostic DEGs, METTL3, HNRNPC and RBM15, were selected in the Lasso regression analysis. When the partial likelihood deviance reached maximum, the value of log (λ) ranged from –3 to –2. At this time, there were two variables whose coefficients did not decay to zero, therefore, these two genes were applied to construct the prognostic model (Supplementary Figure 3). The m6A-related risk score3 = 0.458*RBM15+0.017*HNRNPC.

The risk score of each ACC sample was calculated through m6A risk signature (Figure 4G). According to the median of risk score, 77 ACC samples were divided into high- and low-risk groups. High risk group resulted in worse survival outcome bringing a 38.3% of 5-year OSR, comparing with 64.6% of that in low risk group (Figure 4B). Moreover, high risk levels were tightly associated with undesirable clinicopathological status (Figure 4L), which indicated m6A risk signature was involved in malignant progression of ACC.

Besides, the ROC curves confirmed a great prediction accuracy of m6A risk signature in ACC prognosis, and the accuracy increased slightly with the extension of follow-up time (Figure 4C). In contrast to T, N and clinical stages, m6A risk signature also presented a better prediction accuracy (Figure 4F). PCA and t-SNE analytical results both indicated that the novel risk signature could successfully cluster all ACC samples into two different prognosis groups (Figure 4D–4E). In a word, the m6A risk signature is reliable in theory.

### HNRNPC and RBM15 infrequently mutated in ACC

Using the cBioPortal database, only four out of 89 ACC samples (4.49%) were found to have HNRNPC and RBM15 mutations (Figure 4H). Regarding mutation type, amplification was the most predominant type for all samples (2 cases) and the only type in HNRNPC genetic alteration (Figure 4I–4K). Overall, HNRNPC and RBM15 infrequently mutated in ACC, suggesting that their aberrant expressions may result from epigenetic modification.

### M6A-related risk signature contributes to ACC prognostic analysis

**Figure 5:**
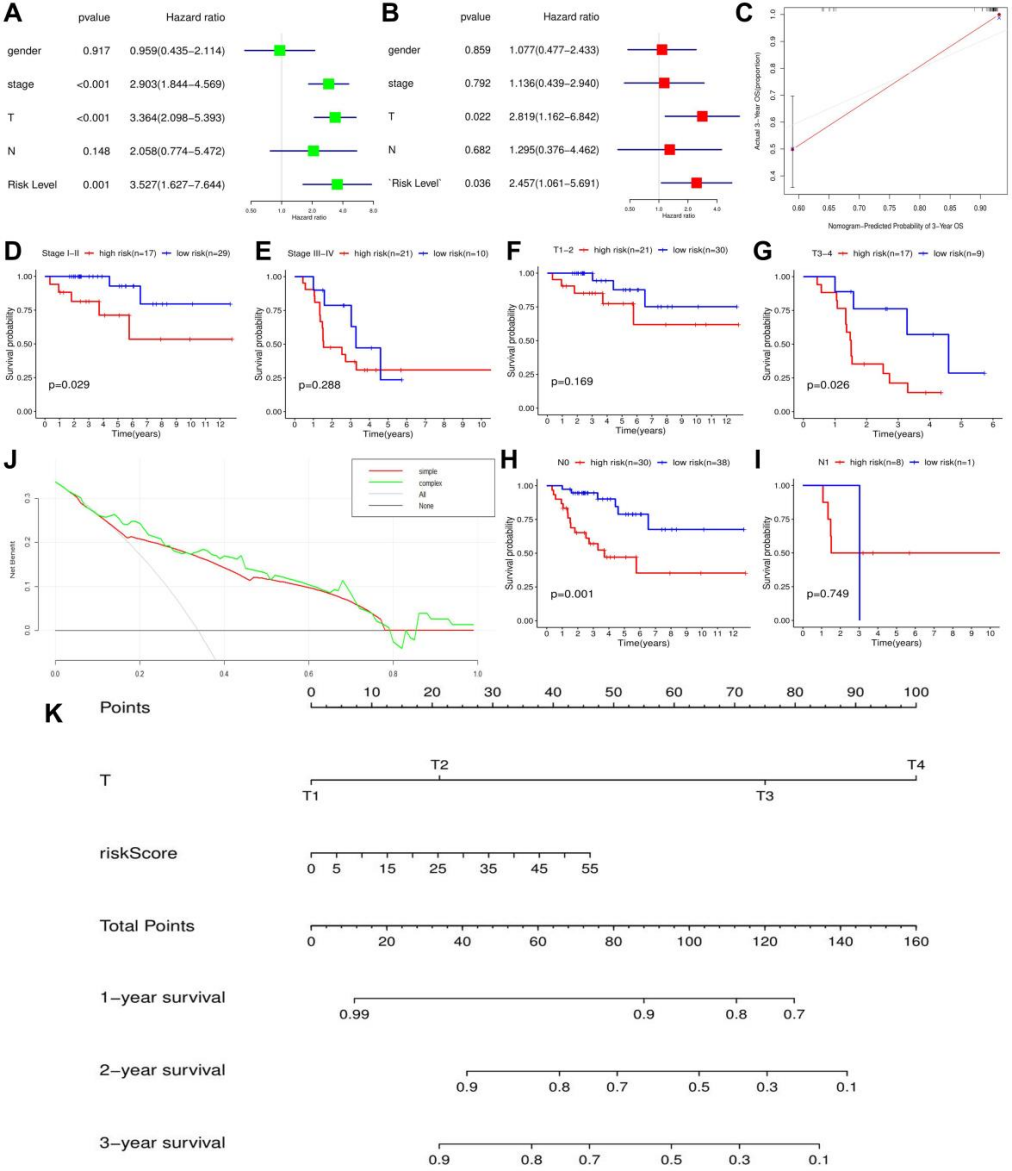
**The effect of m6A-related risk signature on ACC prognosis in TCGA cohort.** (**A**) The results of cox univariate prognostic analysis. (**B**) The results of multivariate prognostic analysis. (**C**) The calibration curves. (**D**–**I**) The prognostic differences of ACC patients with the same clinical subgroups. (**J**) The DCA curve of m6A risk signature. ‘Simple’ curve (red) represents the prognostic model composed of age and clinical stage. ‘Complex’ curve (green) represents the prognostic model composed of age, clinical stage and m6A risk score. (**K**) The nomogram is used to predict the OSR of ACC patient at 1, 2, 3 year. DCA, decision curve analysis.

Based on cox univariate and multivariate analyses, only T stage (*p* = 0.005, HR_mean_ = 3.665) and risk level (*p* = 0.036, HR_mean_ = 2.457) were identified as the independent prognostic factors of ACC (Figure 5A–5B). Meanwhile, the risk signature can distinguish the prognostic difference of patients with Stage I-II (*p* = 0.029), T3-4 (*p* = 0.026) and N0 (*p* = 0.001), but not with Stage III-IV (*p* = 0.288), T1-2 (*p* = 0.169) and N1 (*p* = 0.749) (Figure 5D–5I).

Moreover, when we inserted m6A risk signature in ACC prognostic analysis, the net benefits of clinical decision-making were increased (Figure 5J), which indicated that the novel risk signature can benefit prognostic prediction of ACC. To easily predict the overall survival rate (OSR) of individual at 1,2,3 year, we constructed a nomogram using T stage and m6A risk level, the two independent prognostic factors of ACC (Figure 5K). For example, an ACC patient with T2 stage (20 points) and 35 points of risk score (30 points) will obtain a 55 total points, whose 2-year OSR is predicted to be higher than 80%. Besides, calibration plots showed that the nomogram did well compared with an ideal model (Figure 5C). Altogether, m6A-related risk signature can provide important supplement and new idea for ACC prognostic analysis.

### M6A-related risk signature is confirmed in the validation cohort

**Figure 6:**
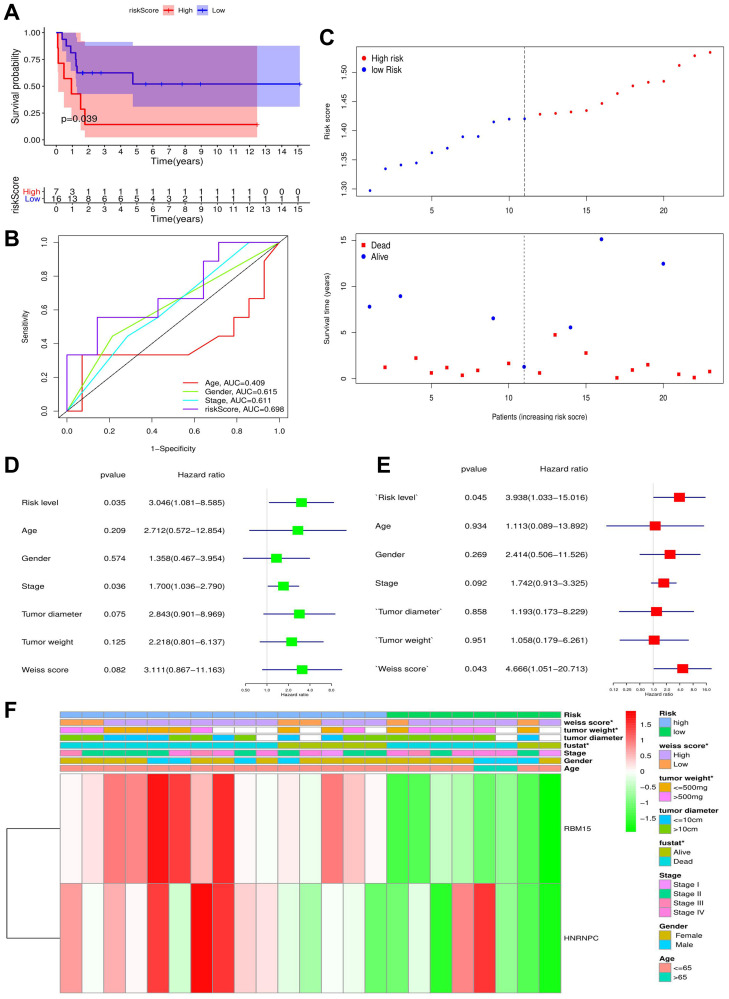
**The validation of m6A-related risk signature in GSE33371 cohort.** (**A**) The survival difference between the high- and low-risk groups. (**B**) Multivariable ROC curves in the validation cohort. (**C**) The risk plots in the validation cohort. (**D**–**E**) identification of independent prognostic factors in the validation cohort. The results of cox univariate regression are shown in green and those of cox multivariate regression are red. (**F**) The heatmap of m6A risk signature in validation cohort. ^*^P < 0.05.

To verify the prognostic value of m6A-related risk signature, GSE33371 was selected as the validation cohort. Similar to training cohort, high risk group led a worse survival outcome (*p* = 0.039) (Figure 6A–6C). Meanwhile, m6A risk signature possessed a better prognostic accuracy than ACC clinicopathological features (AUC = 0.698) (Figure 6B). Moreover, m6A risk level (HR = 3.938, *p* = 0.045) and Weiss score (HR = 4.666, *p* = 0.043) were both identified as ACC-independent prognostic factors (Figure 6D–6E). Besides, high m6A risk was closely with unfavorable tumor weight and Weiss score, but not with tumor diameter and clinical stage (Figure 6F). Altogether, the prognostic value of m6A risk signature was successfully verified in the validation cohort.

### M6A-related risk signature affects immune microenvironment of ACC

**Figure 7:**
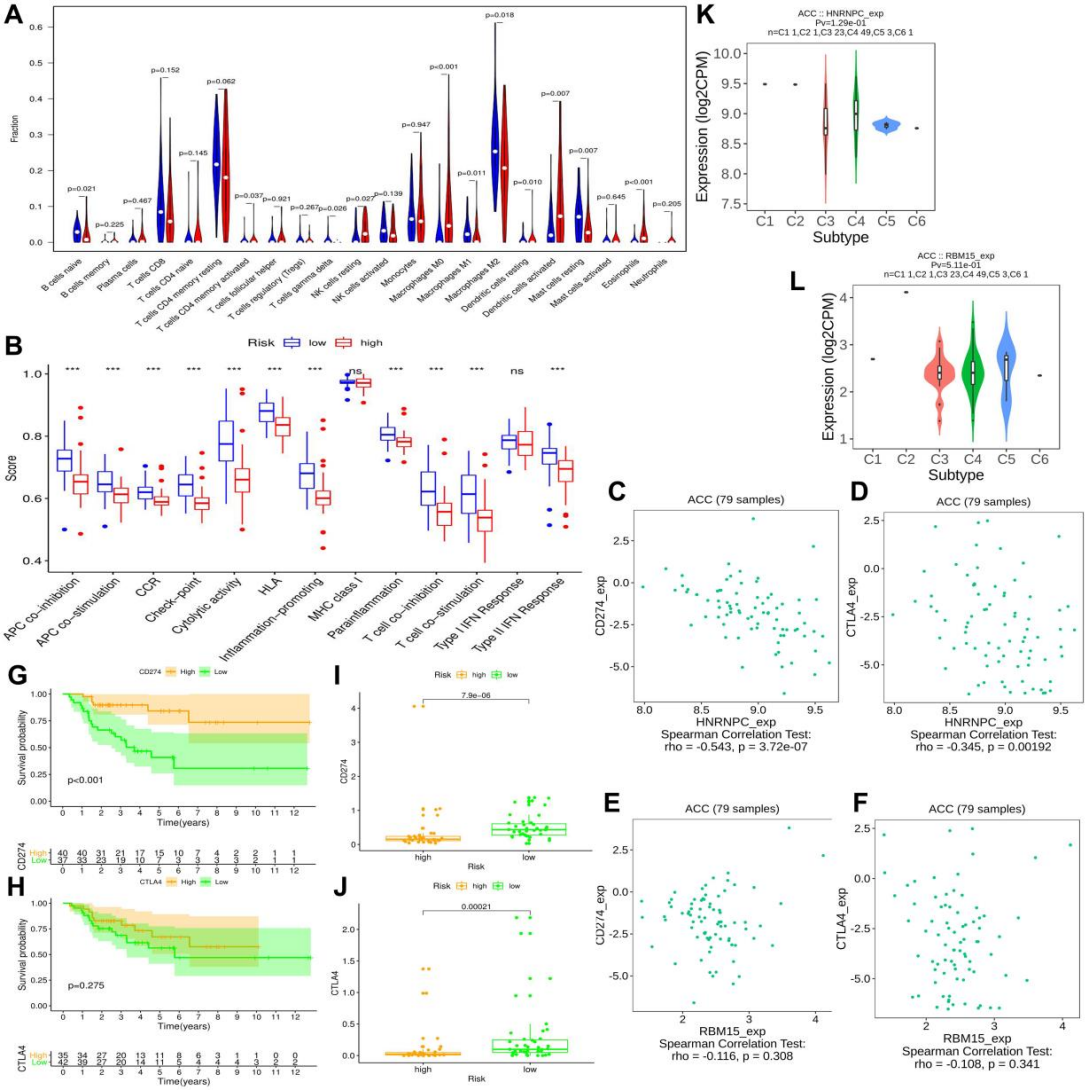
**The effect of m6A-related risk signature on ACC immune microenvironment.** (**A**) Comparison of the infiltrating levels of 22 immune cells between different risk groups. (**B**) Comparison of the activity scores of 13 immune-related pathways between different risk groups. (**C**–**D**) The relationships between HNRNPC expression and CD274 (PD-L1), CTLA4 expressions. (**E**–**F**) The relationships between RBM15 expression and CD274 (PD-L1), CTLA4 expressions. (**G**–**H**) The prognostic differences of ACC patients in TCGA cohort between high- and low-CD274 or CTLA4 expression. (**I**–**J**) The expressive difference of CD274 or CTLA4 between high and low m6A-risk group. (**K**–**L**) The distributions of m6A risk genes in different PAAD immune subtypes. DC, dendritic cell; APC, antigen-presenting cells; CCR, cytokine-cytokine receptor; IFN, interferon.

The immune abundances of 22 leukocyte subtypes in each ACC sample were calculated based on CIBERSORT algorithm (Supplementary Figure 4). Further, we determined the difference of immune cellular proportion between high- and low-risk groups (Figure 7A). High m6A risk can facilitate infiltration levels of T cells CD4 memory activated (*p* = 0.037), NK cells resting (*p* = 0.027), Macrophages M0 (*p* < 0.001), Dendritic cells resting (*p* = 0.010), Dendritic cells activated (*p* = 0.007) and Eosinophils (*p* < 0.001) in ACC; Inversely, it can reduce that of B cells naive (*p* = 0.021), T cells gamma delta (*p* = 0.026), Macrophages M1(*p* = 0.011) Macrophages M2 (*p* = 0.018) and Mast cells resting (*p* = 0.007). Moreover, except for MHC (major histocompatibility complex) class I and Type I IFN (interferon) Response, the activities of other 11 immune pathways were all retarded in high m6A-risk level (Figure 7B).

### High m6A risk level may be unfavorable to ICIs therapy.

Immune checkpoint inhibitors (ICIs) bring an important breakthrough for genitourinary tumors, especially for advanced bladder cancer [31]. Therefore, we further investigated the potential associations between m6A risk level and ICIs therapy. It is now established that the therapeutic effect of ICIs therapy was tightly related to antigen-presenting process [32], immune checkpoint [33] and cytolytic effect [34]. However, these immune processes were all suppressed by high m6A risk level (Figure 7B), suggesting that high m6A risk level may hinder ICIs treatment.

Commonly, patients with PD-L1 overexpression had better response to anti-PD-1 therapy, thus enjoyed better survival [35]. Similar results were observed in this study, ACC patients with high PD-L1 expression (training cohort) had better prognosis than that with low expression (Figure 7G). However, this phenomenon did not appear in CTLA4 (Figure 7H). As for the expressive associations of immune-checkpoint markers with m6A risk genes, the expressions of PD-L1 (CD274) and CTLA4 negatively correlated with that of HNRNPC, while failed to be correlate with that of RBM15 (Figure 7C–7F). And their expressions in high m6A risk group were both lower than those in low risk group (Figure 7I–7J). In a word, high m6A risk not only suppressed immunotherapy-related biological processes, but also repressed the expressions of immune-checkpoint markers. Besides, it was observed that there were no significant differences of HNRNPC and RBM15 expressions between different immune subtypes (Figure 7K–7L).

### HNRNPC promotes the proliferation, migration and invasion of ACC cells.

**Figure 8:**
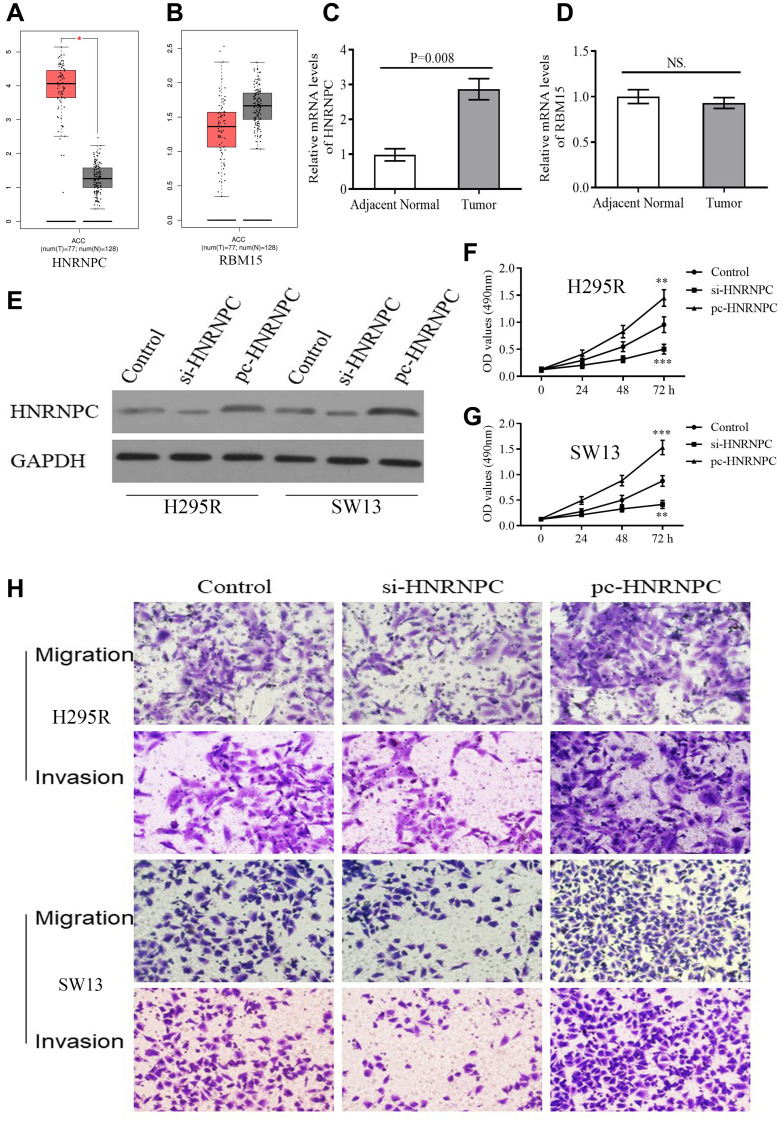
**The expression and biofunction of HNRNPC in ACC.** (**A**–**B**) The differential expression of HNRNPC and RBM15 between tumor and normal samples based on GEPIA database. (**C**–**D**) The differential expression of HNRNPC and RBM15 in 5 pairs of clinical specimens. (**E**) Detection of transfection efficiency. (**F**–**G**) HNRNPC can promote proliferation of H295R and SW13 cells. (**H**) HNRNPC can promote migration and invasion of H295R and SW13 cells. ^*^P < 0.05.

According to GEPIA database, HNRNPC was reconfirmed to be upregulated in tumor samples (Figure 8A). However, there was no expressive difference of RBM15 between tumor and normal samples (Figure 8B). Similarly, overexpression of HNRNPC was detected in clinical tumor tissues by qPCR but not for RBM15 (Figure 8C–8D). Therefore, we focused on the biofunctions of HNRNPC in adrenal cancer cells.

siRNAs and pcDNA 3.1 were used to alter the protein levels of HNRNPC in H295R and SW13 cells. Western blot test revealed that si-HNRNPC can effectively suppress the expression of HNRNPC and pc-HNRNPC can promote it (Figure 8E). Further, silencing HNRNPC can retard the proliferative, migrative and invasive abilities of adrenal cancer cells (Figure 8F, 8G, 8H). Conversely, overexpression of HNRNPC facilitated the proliferation, migration and invasion of adrenal cancer cells (Figure 8F, 8G, 8H). These results reiterated that HNRNPC may serve as an oncogene in ACC.

## DISCUSSION

ACC is a rare urological carcinoma. Although its incidence rate is only 0.7/million to 2.0/million, its 5-year OSR is often less than 30%. Moreover, the treatment of ACC is challenging: the current molecular targeted medicines have not been observably beneficial for patients [36], and even the first-line therapeutic drug, Metortan, has an effective rate of only 23% [37]. Therefore, it is imperative to explore the molecular mechanisms of ACC carcinogenesis and progression. M6A RNA methylation is the most common modification and has been proven to play an important role in tumor progression [38]. Correspondingly, the m6A regulatory genes were also found to be closely associated with AML [39], glioblastoma [19], liver cancer [40], breast cancer [41] and pancreatic cancer [20]. However, the crucial roles of m6A-related genes in ACC have not been fully elucidated. In the present study, we constructed a novel m6A risk signature and demonstrated that the risk signature participated in prognosis, progression and immune microenvironment of ACC by a series of bioinformatic analysis. Further, *in vitro* experiments confirmed the promoting effect of HNRNPC on the proliferation and invasion of ACC cells. These results provide important clues and theoretical foundation for future ACC research.

Expressive difference is the basis for genes to perform their biological functions. According to TCGA database, both RBM15 and HNRNPC from m6A risk signature showed up-regulated in the metastatic ACC samples. However, through the detections of clinical samples, only HNRNPC harbored differential expression between ACC and adjacent normal tissues, while RBM15 showed no ectopic expression. Of note, gene expression possesses spatial and cell-phase specificities [42]. For example, in the early stage of prostate cancer, hASH-1 presented low-expression level before neuroendocrine differentiation occurs [43]. However, when patients progress to neuroendocrine prostate cancer (NEPC), a lethal form of castration-resistant prostate cancer (CRPC), the expression of hASH-1 will be extremely elevated [43]. Besides, given that the inadequacy of clinical specimens in present study, the expression of RBM15 in ACC still requires further validation.

The m6A risk signature was not only an independent prognostic factor in ACC, but also can distinguish the prognostic difference of ACC patients with clinical stage I-II, T3-4 and N0 stages, which brings some inspiration for the ACC clinical system. Firstly, m6A-related risk signature facilitates individualized tumor treatment. Given that there was an obvious difference in 5-year OSR between high- and low-risk groups, the follow-up interval of high risk patients should be appropriately shortened, and some adjuvant therapies should be attempted after adrenalectomy. Secondly, m6A-related risk signature provides an indispensable supplement for ACC prognostic analysis. Current AJCC TNM-stage system for ACC have not shown a survival difference between patients with clinical stage I/II [44]. However, this prognostic difference can be detected by our risk signature (Figure 5D). Comparing with single TNM classification, combination with m6A-related risk signature undoubtedly increases the predicted accuracy for ACC prognosis (Figures 4F and 5J). Thirdly, the nomogram based on T stage and m6A risk score conferred the prediction of OSR straightforward and efficient. In clinical practice, the application of the nomogram may offer more detailed survival information, contributing to make clinical-decision (Figure 5J–5K).

Analyzing the alterations of tumor immune microenvironment is of great importance to find potential therapeutic targets. In present study, m6A-related risk signature can extensively affect the activities of various immune cells and pathways, and resulted in a comprehensive and complex effect on ACC progression. NK cells can kill susceptible tumor cells through perforin-dependent mechanisms or inducing death receptor-mediated apoptosis [45]. The increased proportion of resting NK cells indicates the suppression of the anti-tumor cellular immunity. Macrophages are polarized into M1 and M2 types by different inducers [46]. M1 macrophages induced by interferon (IFN)-γ and TNF-α, possessed promote-inflammatory and cytotoxic antitumor abilities [47]. However, M2 macrophages were commonly responsible for tumor immunosuppression [46]. Therefore, the decreased of Macrophages M1 was also disadvantageous to anti-tumor immunity. Moreover, antigen-presenting cells (APCs), primarily including dendritic cells (DCs) and regulatory T cells (Tregs), can propel anti-cancer immunity by facilitating the release of tumor antigens [48]. The dysfunction of antigen presenting process (Figure 5B) and the ectopic expression of APCs (Figure 5A) both retard the anti-cancer immunity. Besides, except for NK cells, T cells gamma delta are capable of driving potent anti-tumor responses through TCR (T-cell receptor) -mediated cytotoxicity [49]. Hence, decreasing infiltration level of T cells gamma delta also inhibit the anti-cancer immunity (Figure 5A). Notably, almost all immune-related pathways were suppressed in high m6A-risk level (Figure 5B). Sensitive tumor-antigen presenting process (APC co-inhibition and -stimulation, check-point) can activate antigen-specific effector T cells to break away from immunosuppression [50]. Type II interferons (IFN) can activate antitumor M1 Macrophages when it combined with TLR (toll-like receptor) stimulation [51]. CCR (cytokine-cytokine receptor) [52] and T lymphocytes (T cell co-inhibition and -stimulation) are also involved in anti-tumor immunity mediated by some stimulatory cytokines [53]. As a result, deactivation of these immune pathways is commonly undesirable for antitumor process. In a word, the effects of m6A regulators on ACC tumor immunity are extremely complicated, and high m6A-related risk may lead to the suppression of anti-tumor immune effect.

Recently, ICIs have gradually applied to the patients with advanced urothelial carcinoma as first- and second-line therapy [31]. However, only a proportion of patients present therapeutic response and benefit from ICIs treatment. Despite the optimum prediction marker of ICIs therapeutic effect remains controversial, the patients with PD-L1 overexpression or positive commonly have a better objective response rate (ORS) and longer progression-free survival (PFS) [54]. In present study, we found that high m6A risk was accompanied by lower expressions of PD-L1 and CTLA4, meanwhile, it can hinder the activities of pathways referring to immunotherapy (Figure 7B, 7I, 7J). These results indicated that the patients with high m6A risk may fail to benefit from ICIs treatment. It is noteworthy that the blockbuster results about the ICIs therapeutic effect on ACC remain unpublished, and several related trials, such as NCT04187404 and NCT02834013, are still ongoing [55]. Besides, differences in expression thresholds for defining PD-L1 positivity or overexpression make some contradictory results reported [31]. Therefore, further investigation is still needed.

HNRNPC, as a RNA binding protein and m6A ‘Reader’, was proven to bind m6A-containing transcripts and regulate splicing and miRNA maturation [56]. Although the elevated expression of HNRNPC was incidentally observed in some cancers, including glioblastoma [57], hepatocellular carcinoma [58] and melanoma [59], the molecular mechanism of HNRNPC in carcinogenesis has been poorly elaborated. In the present study, HNRNPC knockdown inhibited the proliferation, migration and invasion of ACC cells, which was the first verification of its biofunction in ACC. In breast cancer cells, Wu YS *et al*. ascertained that the repression of HNRNPC inhibited cell proliferation and tumor growth through the accumulation of endogenous dsRNA (double-stranded RNA) and the activation of down-stream interferon (IFN) response [60]. Similarly, we also found that high m6A-risk level can suppress the activity of Type II IFN response (Figure7B), which suggested that HNRNPC may also promote ACC progression via regulating the IFN response.

Naturally, there are some limitations in this study. Firstly, the clinical and TCGA sample sizes are insufficient. However, due to the rare incidence rate and the controversy of surgical indications for metastatic cases [61], it is indeed tough to obtain ACC data with a satisfactory sample size. Secondly, the ACC clinical information from TCGA was not comprehensive, some pivotal clinical features, such as age, histopathological grade and M stage, have not been included in bioinformatic analyses. Thirdly, we did not conduct *in vivo* experiments to confirm the role of HNRNPC in ACC progression. Fourthly, although the prognostic value of m6A risk signature has been validated in GSE33371 dataset, it remains to be tested in real clinical cohort.

## CONCLUSIONS

Based on TCGA and GEO database, we constructed a novel m6A-related risk signature consisting of RBM15 and HNRNPC, and verified its prognostic value in the validation cohort (GSE33371). The risk signature was not only beneficial for ACC prognostic analysis, but also can affect the immune microenvironment. Further, *in vitro* experiments confirmed the oncogenic role of HNRNPC in ACC cells. In conclusion, m6A regulatory genes, especially HNRNPC, may have a profound impact on the malignant progression and prognosis of ACC.

## Supplementary Materials

Supplementary Figures

Supplementary Tables
