# Supplementary material for: Bioinformatic analyses and experimental validation of the role of m6A RNA methylation regulators in progression and prognosis of adrenocortical carcinoma
Source: Aging (Albany NY). 2021 Apr 21;13(8):11919–41. doi: 10.18632/aging.202896 (PMC8109058; doi:10.18632/aging.202896)
Supplement: Supplementary Figures [file aging-13-202896-s001.pdf]

SUPPLEMENTARY FIGURES

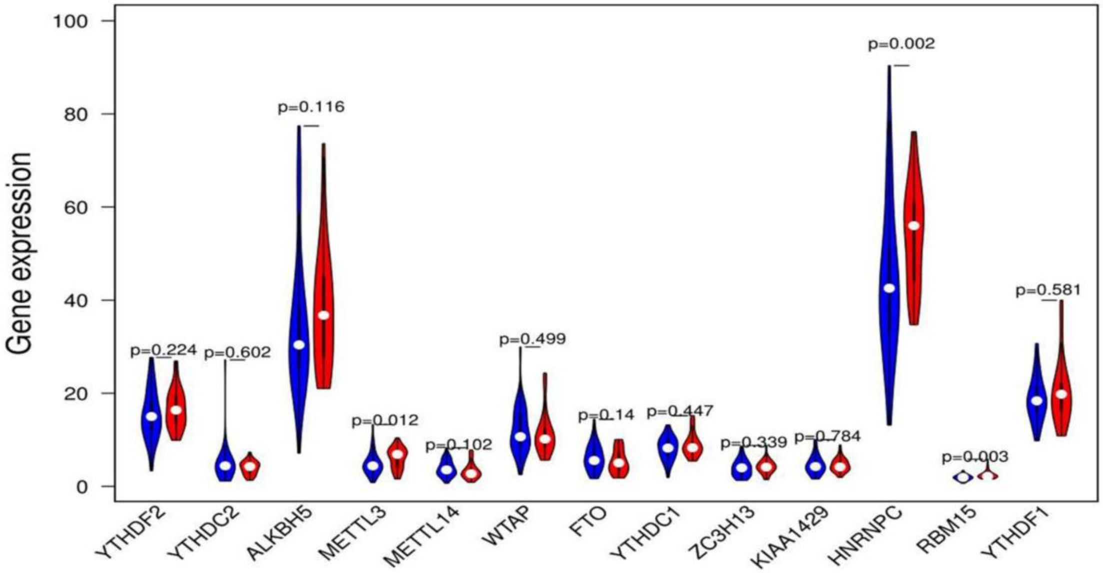

Supplementary Figure 1. The violin plot of differential expression of m6A genes between localized and metastatic tumor.

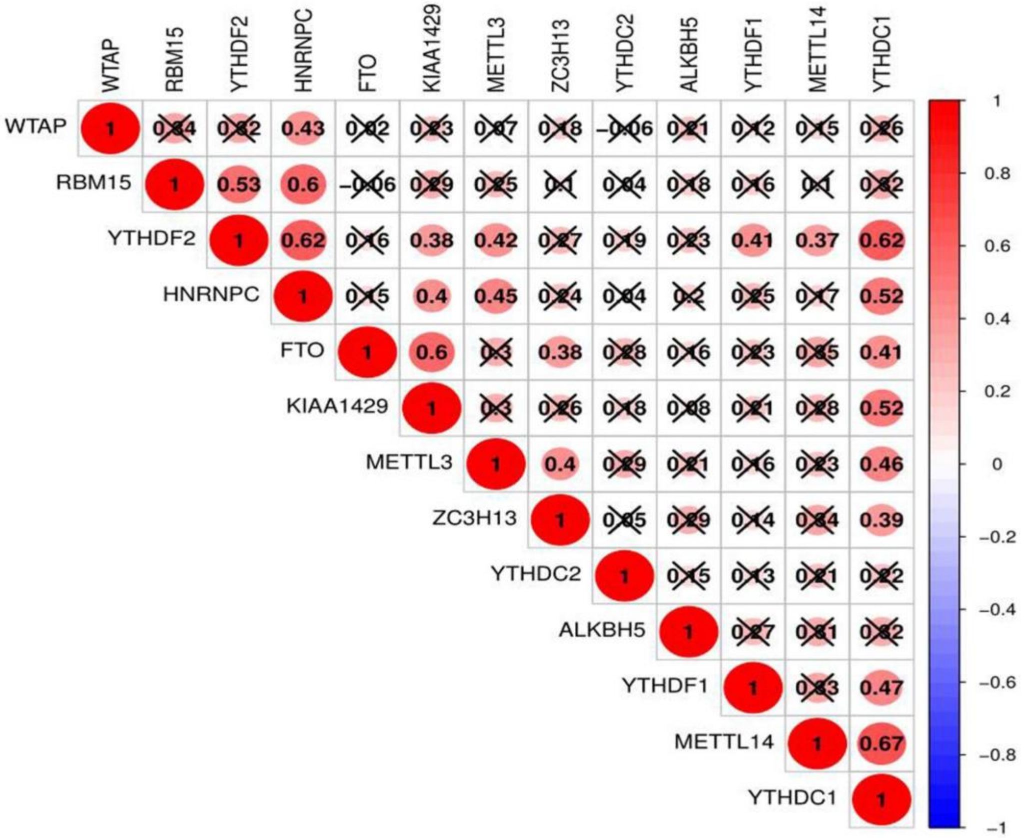

Supplementary Figure 2. Correlation analysis among m6A regulatory genes. The cross signal (X) indicates no statistical significance. Positive and negative correlations are shown in red and blue, respectively. A darker color of circle indicates a stronger correlation.

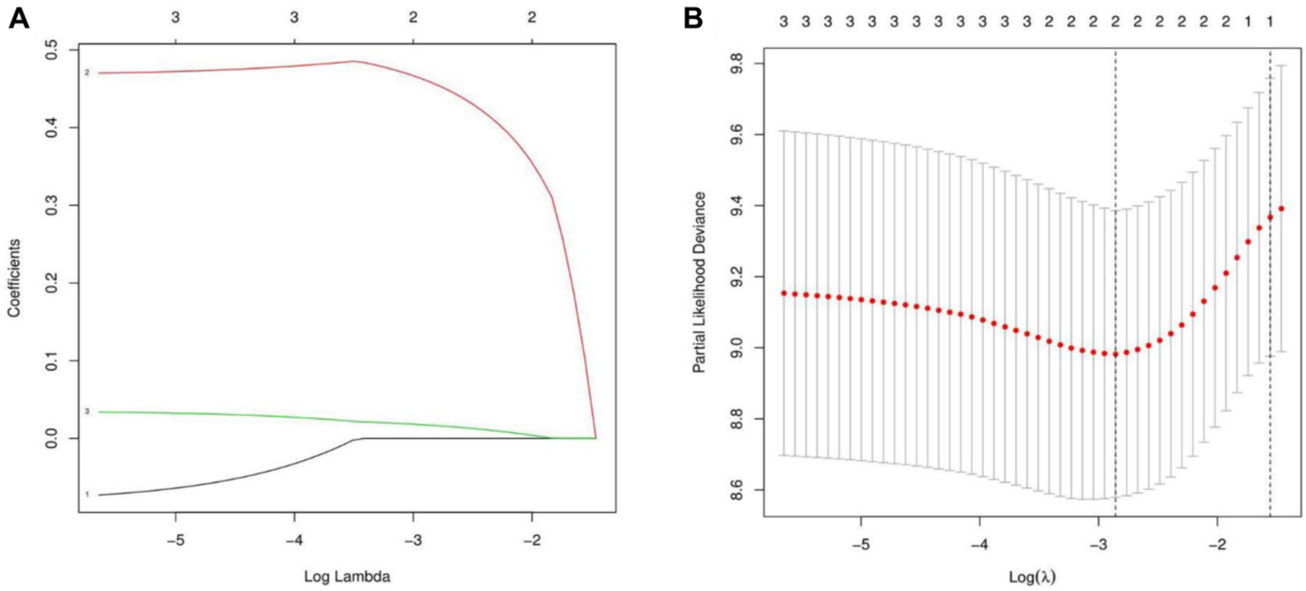

Supplementary Figure 3. The process of Lasso regression analysis.

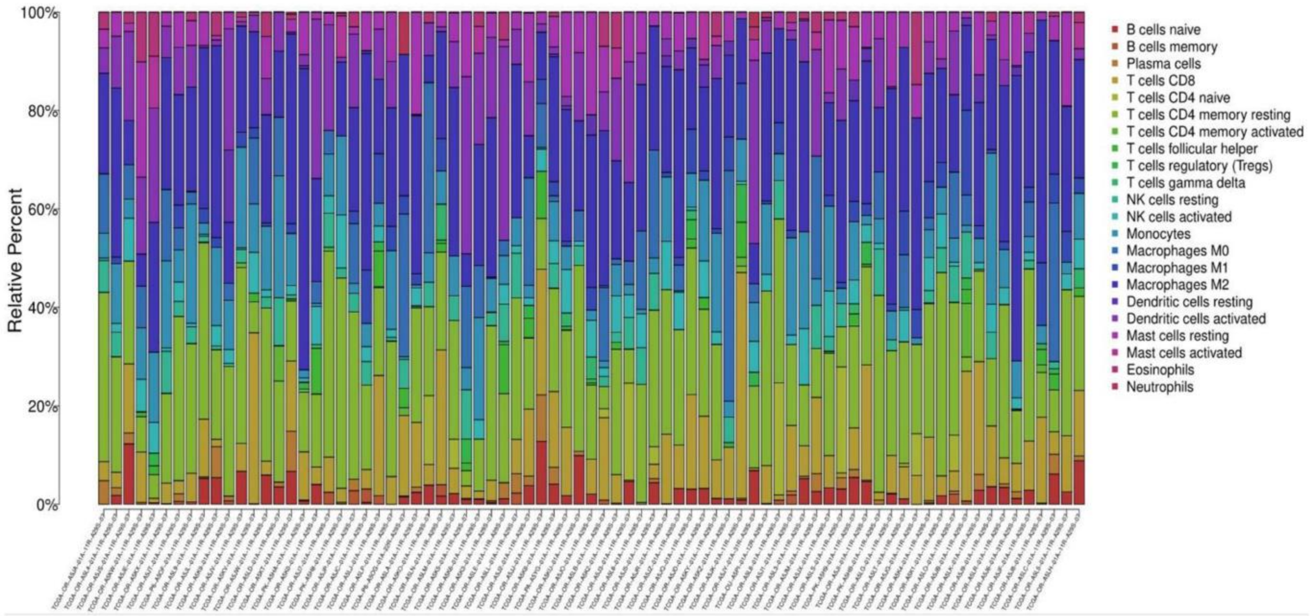

Supplementary Figure 4. The landscape of immune abundance of 22 leukocyte subtypes in each ACC samples. ACC, Adrenocortical carcinoma.
