# Supplementary material for: Bioinformatic analyses and experimental validation of the role of m6A RNA methylation regulators in progression and prognosis of adrenocortical carcinoma
Source: Aging (Albany NY). 2021 Apr 21;13(8):11919–41. doi: 10.18632/aging.202896 (PMC8109058; doi:10.18632/aging.202896)
Supplement: Supplementary Tables [file aging-13-202896-s002.pdf]

SUPPLEMENTARY TABLES

Supplementary Table 1. Filtering settings of TCGA data.

Filter Items	Gene Expression Data	Clinical Data
Primary Site	Adrenal gland	Adrenal gland
Program	TCGA	TCGA
Project	TCGA-ACC	TCGA-ACC
Disease type	Adenomas and adenocarcinomas	Adenomas and adenocarcinomas
Data category	Transcriptome profiling	Clinical
Data type	Gene Expression Quantification	Clinical supplement
Experimental Strategy	RNA-seq	—
Workflow type	HTSeq-FPKM	Bcr-xml

Abbreviations: Seq, sequence; FPKM, fragments per kilobase per million; Bcr, breakpoint cluster region; xml, extensible markup language.

Supplementary Table 2. Primers list.

Gene	Primer	Sequence (5′->3′)
HNRNPC	Forward	CCCTTCTCCGTCCCCTCTAC
	Reverse	CCCGAGCAATAGGAGGAGGA
RBM15	Forward	GTGAGGACTCGACTTCCCG
	Reverse	GCCGCTATCGGTCTTTCCG
si-HNRNPC	Primer	AAAAAAUCUCACAAGAAGGGG
